# Transcriptional profiling of HOXA9-regulated genes in human glioblastoma cell models

**DOI:** 10.1016/j.gdata.2015.05.010

**Published:** 2015-05-19

**Authors:** Céline S. Gonçalves, Ana Xavier-Magalhães, Marta Pojo, Ana Isabel Oliveira, Sara Correia, Rui M. Reis, Nuno Sousa, Miguel Rocha, Bruno M. Costa

**Affiliations:** a Life and Health Sciences Research Institute (ICVS), School of Health Sciences, University of Minho, Braga, Portugal; b ICVS/3B's—PT Government Associate Laboratory, Braga/Guimarães, Portugal; c Centre of Biological Engineering, Department of Informatics, University of Minho, Campus de Gualtar, 4710-057 Braga, Portugal; d Barretos Cancer Hospital, Molecular Oncology Research Center, Rua Antenor Duarte Vilela, 1331-Doutor Paulo Prata, Barretos, SP 14780-000, Brazil

**Keywords:** HOXA9, Transcriptome, GBM, Microarrays, R/bioconductor

## Abstract

The data here described pertain to the article by Pojo et al. (2015) [10] titled “A transcriptomic signature mediated by HOXA9 promotes human glioblastoma initiation, aggressiveness and resistance to temozolomide” (Pojo et al., 2015 [10]). *HOX* genes are part of the homeobox gene family, which encodes transcription factors crucial during embryonic development (Grier et al., 2005; Pearson et al., 2005 [6], [9]) and also in postdevelopmental regulation (Neville et al., 2002; Yamamoto et al., 2003; Takahashi et al., 2004; Morgan 2006 [8], [14], [13], [7]). Alterations interfering with the regulation of these genes may lead to tumorigenesis in adults. Due to their contributions in the control of important cellular processes, the deregulation of *HOX* genes is ultimately correlated with cancer treatment failure and patients' poor prognosis (Golub et al., 1999; Abdel-Fattah et al., 2006 [5], [1]; Costa et al., 2010 [4]; Pojo et al., 2015 [10]). Recently, our studies showed that *HOXA9* overexpression is associated with poor prognosis in patients with glioblastoma (GBM), the most common and most malignant primary brain tumor. Mechanistically, HOXA9 is associated with resistance to chemotherapy and with pro-proliferative, pro-invasive and anti-apoptotic features (Costa et al., 2010 [4]; Pojo et al., 2015 [10]) in GBM in vitro models. Since HOXA9 is a transcription factor, its target genes can be the true biological effectors of its aggressiveness. In this context, whole genome Agilent's microarrays were used to obtain the full transcriptome of HOXA9 in a variety of GBM cell models, including human immortalized astrocytes, established GBM cell lines, and GBM patient-derived cell cultures. Here, we provide detailed methods, including experimental design and microarray data analyses, which can be accessed in Gene Expression Omnibus (GEO) under the accession number GSE56517. Additional interpretation of the data is included and supplemented in (Pojo et al., 2015 [10]).

**Table:** 

Specifications	
Organism/cell line/tissue	*Homo sapiens*/immortalized astrocytes; glioblastoma cell lines; and glioblastoma patient-derived cells
Sex	Male for U251, GBML18, and U87MG; unknown for hTERT/E6/E7
Sequencer or array type	Whole Human Genome Microarray (G4112F, 4x44K; Agilent Technologies)
Data format	Raw and processed
Experimental factors	HOXA9 overexpression or silencing
Experimental features	We performed microarrays analysis on cells with different mRNA levels of HOXA9 to determine its full transcriptome.
Consent	N/A
Sample source location	ICVS, University of Minho, Braga, Portugal

## 1 Direct link to deposited data

http://www.ncbi.nlm.nih.gov/geo/query/acc.cgi?acc=GSE56517.

## 2 Experimental design, materials and methods

### 2.1 Tissue culture

For this study, 4 different cell lines were used: 2 established human glioblastoma (GBM) cell lines, U87MG and U251MG, purchased from American Type Culture Collection (ATCC®); 1 cell line of human immortalized astrocytes, hTERT/E6/E7, kindly originally supplied by Dr. Russell Pieper; and 1 primary GBM culture, GBML18, established in our lab from a clinical specimen. All cell lines were cultured in Dulbecco's Modified Eagle Medium (DMEM; Gibco®) supplemented with 10% fetal bovine serum (FBS; Biochrom GmbH) and 1% penicillin–streptomycin (Invitrogen™). Cells were maintained in a humidified atmosphere at 37 °C and 5% (v/v) CO_2_.

### 2.2 Cell transfections and transductions

U87MG and hTERT/E6/E7 cells, which do not express detectable levels of *HOXA9* mRNA, were previously retrovirally infected with murine stem cell virus (MSCV) containing the HOXA9 coding region to overexpress this gene (U87MG-HOXA9 and hTERT/E6/E7-HOXA9) or with an empty vector (U87MG-MSCV and hTERT/E6/E7-MSCV, control) [4]. Selection of transfected cells was performed using 500 ng/μl of G418 (Sigma-Aldrich®). U251MG and GBML18 cells, which present high levels of *HOXA9* mRNA, were also previously transfected with a pGFP-V-RS plasmid (TG307647, clones GI330583 and GI330584; Origene Technologies, Inc.) containing *HOXA9*-specific shRNAs (U251-shHOXA9 and GBML18-shHOXA9) or non-effective shRNA sequences (U251-shCtrl and GBML18-shCtrl) [10]. Selection of transfected cells was performed for 3-weeks, using 0.5 μg/ml of puromycin (Santa Cruz Biotechnology®).

### 2.3 RNA isolation, purification and cDNA quality control analysis

Total RNA was isolated from 8 × 10^5^–1 × 10^6^ exponentially growing cells from a T75-flask. The TRIzol method (Invitrogen™) was used to extract the total cellular RNA from the 8 cell lines previously obtained (3 replicates from each cell line). Extracted RNA was purified using the RNeasy Plus Micro kit (Qiagen®) and quantified using the Nanodrop 2000 (under the Nucleic Acid option; Thermo Scientific, Inc.; Table 1). RNA quantification, integrity and purity were also validated using the Agilent 2100 bioanalyzer (Agilent Technologies; Table 1). All RNA samples presented A260/A280 ratios above 1.8, electropherograms with 2 distinct peaks, corresponding to the 18S and 28S ribosomal RNA, and RNA integrity numbers (RIN) above 8, as recommended for microarray analysis.

To validate that the obtained samples were a good model of *HOXA9* mRNA levels modulation, *HOXA9* overexpression or silencing was confirmed. To do so, 1 μg of total RNA was reverse transcribed using the High Capacity cDNA Reverse Transcriptase kit (Alfagene®), and *HOXA9* quantitative PCR was performed. This cDNA was further used to validate the results obtained by microarrays.

### 2.4 Microarray experiments and gene expression analysis

#### 2.4.1 cRNA preparation, labeling, purification and quality control analysis

Complementary RNA (cRNA) was obtained using the Low Input Quick Amp Labeling kit, One-Color (Agilent Technologies) using 200 ng of the total RNA. RNA samples were labeled with Cyanine 3-CTP and amplified together with Agilent One Color Spike-In controls, which were used as positive controls to monitor the amplification, labeling and microarray scanning. Labeled/amplified RNA was purified using the RNeasy Mini Kit (Qiagen®) according to the manufacturer's instructions. Nanodrop 2000 (under the Microarray measurement option; Thermo Scientific, Inc.) was used to obtain the cRNA and the Cyanine 3 dye concentrations, and to verify the quality of the cRNA (A260/280 ratio; Table 2). All samples presented A260/280 ratios between 2.14 and 2.33, cRNA yield > 6.08 and specific activity > 8.1 (recommended to be above 1.8, 1.65 and 6, respectively).

#### 2.4.2 Hybridization and washing

Labeled cRNA (1.65 μg for all conditions) was mixed according to the manufacturer's protocol and hybridized in a Whole Human Genome Microarray (G4112F, 4x44K; Agilent Technologies) at 65 °C for 17 h, under 10 rpm using a hybridization rotator (Agilent Technologies). After hybridization, slides were disassembled in Wash Buffer 1 and washed once with Wash Buffer 1 at room temperature and once with Wash Buffer 2 pre-warmed overnight at 37 °C according to the manufacturer's instructions.

#### 2.4.3 Scanning and feature extraction

Slides were immediately scanned using the DNA Microarray Scanner with SureScan High-Resolution Technology (Agilent Technologies) using 5 different Green PMT gains (100%, 80%, 60%, 40% and 20%). The feature extraction was performed using the grid 014850_D_20070207 and the protocol GE1_107_Sep09 from Agilent. To choose the best scanning, several parameters of the feature extraction were taken into account, such as the number of saturated spikes and probes, the shape of the histogram of signals plot, the Agilent spike-in plot and the evaluation metrics. Scannings with lower number or no saturated spikes and probes, with higher number of good metrics and with the better histogram shape and spike-in plot were used for subsequent analyses.

### 2.5 R workflow for data processing and analysis

For gene expression microarray data processing and analysis, the *limma* [11] and *hgug4112a.db* [3] packages of the Bioconductor software platform (http://www.bioconductor.org) were used. The complete script run for each cell line is available in the Supplementary material, being here given some excerpts to illustrate the main data processing and analysis steps.

#### 2.5.1 Pre-processing data

After loading the microarrays raw data, a pre-processing was performed through background correction (using the *normexp* method), normalization between arrays using quantile normalization and log2 transformation. Probes representing control spots were removed, keeping only the ones with status equal to “Gene”. The expression values for replicated probes with the same *ProbeName* were averaged. These steps are shown in the following script, which was run for all cell lines. The last line in the script is used to save files with processed data to be submitted to GEO.

RG = read.maimages(targets, columns = …)

RG = backgroundCorrect(RG, method ="normexp", offset = 1)

RG$G = normalizeBetweenArrays(RG$G, method ="quantile")

RG$G = log2(RG$G)

spottypes = readSpotTypes();

RG$genes$Status = controlStatus(spottypes,RG)

i = RG$genes$Status =="Gene"

RG.nocontrol = RG[i,]

E = new("MAList", list(targets = RG.nocontrol$targets, genes = RG.nocontrol$genes, source = RG.nocontrol$source, M = RG.nocontrol$Gb, A = RG.nocontrol$G))

E.avg = avereps(E, ID = E$genes$ProbeName)

write.table(E.avg$A, "processed-cellLine.csv", sep = ",")

#### 2.5.2 Annotation

The mapping between probe identifiers and gene symbols was done using the annotation provided by Agilent (http://www.chem.agilent.com/cag/bsp/gene_lists.asp) and the *hgug4112a.db* annotation package from Bioconductor [3], which provides detailed information about the hgug4112a platform. For each probe identifier, the consensus gene symbol and description were retrieved from Bioconductor packages if the annotation exists, otherwise the Agilent annotation was assumed.

#### 2.5.3 Differential expression

To find the differentially expressed transcripts between each pair of conditions for a given cell line (HOXA9-high vs HOXA9-low expressing cells), the *lmFit* function from the *limma* Bioconductor package was used to fit a linear model. Next, relevant statistics were calculated using the Empirical Bayes method. Finally, the transcripts were ranked according to their adjusted p-values, where false discovery rates were controlled by the BH (Benjamini and Hochberg) method [2] to address the issues related to multiple testing. Significance was considered for adjusted p-values < 0.05.

f = factor(targets$Condition, levels = unique(targets$Condition))

design = model.matrix(~ 0 + f)

colnames(design) = levels(f)

contrast.matrix = makeContrasts("A_HOXA9-A_control", levels = design)

fit = lmFit(E.avg$A, design)

fit2 = contrasts.fit(fit, contrast.matrix)

fit2 = eBayes(fit2)

output = topTable(fit2, adjust ="BH", coef ="A_HOXA9-A_control",

genelist = E.avg$genes, number = 5000, p.value = 0.05)

output_allgenes = topTable(fit2, adjust ="BH",

coef ="A_HOXA9-A_control",

genelist = E.avg$genes, number = 41000)

#### 2.5.4 Intersection

The results of differential expression for each paired cell line (hTERT/E6/E7-MSCV vs. hTERT/E6/E7-HOXA9; U87MG-MSCV vs. U87MG-HOXA9, U251-shCtrl vs. U251-shHOXA9, and GBML18-shCtrl vs. GBML18-shHOXA9) were used to find transcripts consistently differentially expressed in all cell lines, by computing the intersection of these sets.

overCellLineA = output[output$logFC > 0,]

underCellLineA = output[output$logFC < 0,]

#intersection of overexpression genes from 2 cell lines

intersectOver = intersect(rownames(overCellLineA), rownames(overCellLineB))

over = output[intersectOver,1:8]

over$p.value.A = output[intersectOver,13]

over$p.value.B = output_B[intersectOver,13]

over$logFC.A = output[intersectOver,9]

over$logFC.B = output_B[intersectOver,9]

### 2.6 Validation of microarray data by PCR

After the identification of the differentially expressed genes due to *HOXA9* modulation, reverse transcriptase PCR (RT-PCR or qRT-PCR) analyses were performed to validate the microarray data in a subset of the differentially expressed target genes [10]. Specifically, selected genes for validation were *RAC2*, *CXCL1*, *NDRG1* and *TOX2* for hTERT/E6/E7 cells; *ICAM2*, *BAMBI*, *ANGPT2* and *PDGFRB* for U87MG cells; *TOX2*, *NDRG1*, *RAC2* and *NPR3* for GBML18 cells; and *C10orf10*, *PDGFRB*, *DKK1* and *SOX2* for U251MG cells.

### 2.7 Functional enrichment analysis

**Fig. 1:**
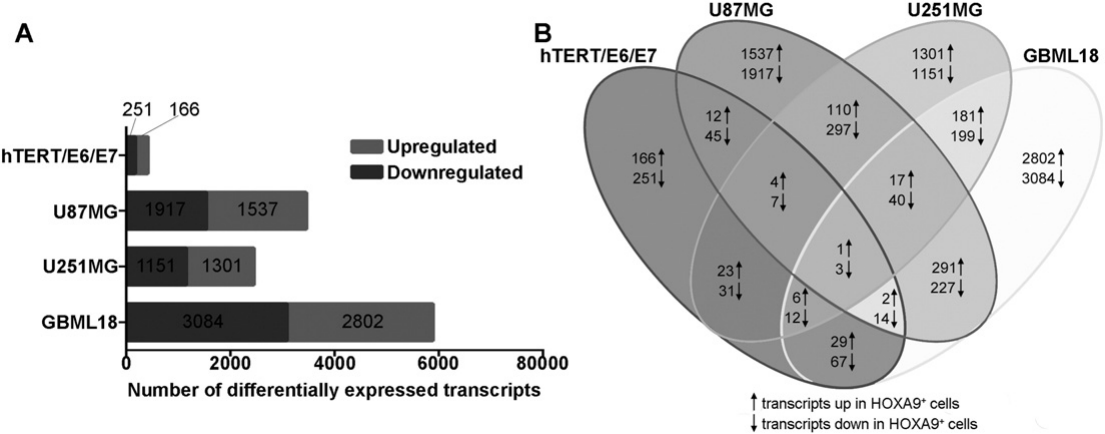
A) Graph representing the number of differentially expressed transcripts in all tested GBM cell models. Transcripts upregulated and downregulated are those whose expression is significantly increased or decreased, respectively, in the presence of *HOXA9*. B) Venn diagram summarizing the number of differentially expressed transcripts upon *HOXA9* modulation in each cell line. In each area, the total number of transcripts within each intersection is represented.

As reported in [10], due to *HOXA9* expression (GEO accession number GSE56517), a total of 417 probes were significantly differentially expressed in hTERT/E6/E7 cells (166 upregulated and 251 downregulated); 3454 probes in U87MG cells (1537 upregulated and 1917 downregulated); 2452 probes in U251MG cells (1301 upregulated and 1151 downregulated); and 5886 probes in GBML18 patient-derived primary cells (2802 upregulated and 3084 downregulated; Fig. 1A). In this context, GBML18 cells were the ones with the highest number of differentially expressed transcripts, followed by U87MG, U251MG and hTERT/E6/E7. The genes *EMILIN2* (upregulated in the presence of HOXA9), and *MME*, *DIRAS1* and *AGPAT3* (downregulated in the presence of HOXA9), whose roles in glioma were not yet studied, were common to the 4 GBM models (Fig. 1B). Even though, the number of genes consistently regulated by HOXA9 increases when comparing only the GBM cells (57 common transcripts; 17 upregulated and 40 downregulated). These results suggest that the transcriptome of HOXA9 is cell-type dependent.

In order to integrate the differentially-expressed genes in biologically relevant groups, they were used for Database for Annotation, Visualization and Integrated Discovery (DAVID) analyses and displayed in KEGG, GO, or Reactome pathways, or used for gene set enrichment analysis (GSEA; http://www.broad.mit.edu/gsea/). For GSEA analysis [12], gene sets databases from MSigDB C2 collection version 3 were used (available online). The permutation type used was “gene sets”, while the default option was used for all other parameters. Only results with a p-value < 0.05 (for DAVID) or a false discovery rate < 0.25 (for GSEA) were considered significant. The obtained results were already published in [10]. Briefly, relevant pathways related to cellular adhesion and migration, cell cycle, DNA repair and replication, RNA processing, stem-cell phenotype, vasculature development, and immune-related pathways were shown to be enriched in the HOXA9 transcriptome. These features are known as important cancer hallmarks that influence the tumorigenic process, suggesting the putative importance of *HOXA9* for GBM development, progression, and aggressiveness [10].

## 3 Discussion

Herein, we describe the transcriptome of HOXA9 in human immortalized astrocytes (hTERT/E6/E7), GBM cell lines (U87MG and U251MG) and in a patient-derived GBM cell culture (GBML18). These data include *HOXA9*-overexpression models (hTERT/E6/E7 and U87MG) and *HOXA9*-silencing models (U251MG and GBML18). *HOXA9*-overexpression augmented the expression of genes associated with increased stem-cell characteristics, invasion, migration, and tumor vasculature, among other cancer hallmarks. Concordantly, *HOXA9*-silencing decreased the expression of genes associated with the same characteristics. Collectively, these data suggests the importance of *HOXA9* in GBM and may explain the poor survival of patients overexpressing this gene [4], [10].

## Conflict of interest

The authors declare no conflict of interests.

## Figures and Tables

**Table 1:** RNA purity and quality assessment for microarray experiments

	Sample	Nanodrop		Bioanalyzer
A260/280^a^	A260/230^a^	RIN^b^
hTERT/E6/E7	Control #1	2.12	1.90	9.1
HOXA9 #1	2.11	1.89	9.6
Control #2	2.10	2.03	9.9
HOXA9 #2	2.09	2.17	9.4
Control #3	2.10	2.22	9.3
HOXA9 #3	2.07	2.19	9.6
U87MG	Control #1	2.07	2.16	9.6
HOXA9 #1	2.09	1.91	9.5
Control #2	2.10	2.16	9.3
HOXA9 #2	2.08	1.87	9.1
Control #3	2.11	2.17	9.4
HOXA9 #3	2.12	2.20	9.5
U251	shCtrl #1	2.09	2.22	9.1
shHOXA9 #1	2.11	2.03	9.3
shCtrl #2	2.08	2.02	9.5
shHOXA9 #2	2.09	2.09	9.4
shCtrl #3	2.09	1.94	9.6
shHOXA9 #3	2.10	2.06	9.1
GBML18	shCtrl #1	2.05	1.99	9.9
shHOXA9 #1	2.10	2.05	9.6
shCtrl #2	2.07	1.81	8.0
shHOXA9 #2	2.04	2.13	9.6
shCtrl #3	2.14	2.23	9.6
shHOXA9 #3	2.13	2.18	9.8

a Nucleic acid is detected at 260 nm, whereas proteins, salts and solvents are detected at 280 and 230 nm. Thus, ratios demonstrate if the RNA is devoid of these contaminants. For microarray experiments, ratios should be ≥ 1.8;

b RIN = RNA integrity number, and provides a quantitative value for RNA integrity that facilitates the standardization of quality interpretation. For microarray experiments, RIN should be ≥ 7.

**Table 2:** cRNA quality, yield and specific activity assessment for microarray experiments

	Sample	Nanodrop				
A260/280^a^	[Cyanine 3 dye] (pmol/μl)	[cRNA] (ng/μl)	cRNA yield^b^ (μg)	Specific activity^c^
hTERT/E6/E7	Control #1	2.33	3.5	387.9	11.6	9.0
HOXA9 #1	2.30	4.8	487.4	14.6	9.9
Control #2	2.23	7.2	551.7	16.6	13.1
HOXA9 #2	2.23	7.0	552.3	16.6	12.7
Control #3	2.24	5.4	445.9	13.4	12.0
HOXA9 #3	2.22	5.6	457.9	13.7	12.1
U87MG	Control #1	2.30	3.6	402.6	12.1	8.90
HOXA9 #1	2.32	2.8	348.4	10.5	8.1
Control #2	2.27	5.8	383.8	11.5	15.2
HOXA9 #2	2.26	7.2	476.8	14.3	15.2
Control #3	2.17	9.4	558.5	16.8	16.9
HOXA9 #3	2.23	8.5	555.4	16.7	15.3
U251	shCtrl #1	2.24	8.7	261.4	7.84	33.3
shHOXA9 #1	2.21	9.9	299.0	8.97	33.1
shCtrl #2	2.14	6.1	213.6	6.41	28.6
shHOXA9 #2	2.18	7.5	237.2	7.12	31.6
shCtrl #3	2.20	12.1	266.7	8.00	45.4
shHOXA9 #3	2.22	10.1	202.8	6.08	49.8
GBML18	shCtrl #1	2.22	5.8	226.9	6.81	25.6
shHOXA9 #1	2.22	9.5	367.6	11.0	25.8
shCtrl #2	2.18	5.1	205.0	6.15	24.9
shHOXA9 #2	2.24	5.8	245.1	7.35	23.7
shCtrl #3	2.22	10.8	306.3	9.19	35.3
shHOXA9 #3	2.20	8.8	262.4	7.87	33.5

a Nucleic acid is detected at 260 nm, whereas proteins, salts and solvents are detected at 280 and 230 nm.

b cRNA yield = ([cRNA] × elution volume) / 1000; should be > 1.65.

c Specific activity = ([Cy3] / [cRNA]) × 1000; should be > 6.
